# Dexmedetomidine Blocks the ERK Pathway by Inhibiting MAP3K8 to Achieve a Protective Effect in Lung Ischemia/Reperfusion Injury

**DOI:** 10.1002/kjm2.70045

**Published:** 2025-05-15

**Authors:** Chun‐Huan Hu, Ru Qian, Yong‐Bo Wang, Lian‐Di Li, Chun‐Xing Miao

**Affiliations:** ^1^ Department of Anesthesiology The Second Affiliated Hospital of Mudanjiang Medical University Mudanjiang Heilongjiang People's Republic of China; ^2^ Department of Critical Medicine The Second Affiliated Hospital of Mudanjiang Medical University Mudanjiang Heilongjiang People's Republic of China; ^3^ Department of Respiratory Medicine The Second Affiliated Hospital of Mudanjiang Medical University Mudanjiang Heilongjiang People's Republic of China; ^4^ Department of Chest Surgery The Second Affiliated Hospital of Mudanjiang Medical University Mudanjiang Heilongjiang People's Republic of China

**Keywords:** dexmedetomidine, ERK pathway, inflammation, lung ischemia/reperfusion injury, MAP3K8

## Abstract

Lung ischemia/reperfusion injury (LIRI) is a primary contributor to morbidity and mortality following lung transplantation. Dexmedetomidine (DEX) protects the lungs from I/R injury, but the underlying mechanisms remain uncertain. This paper examined the protective effect of DEX in LIRI and elucidated the potential regulation involved. LIRI was induced in mice, followed by the detection of pulmonary arterial pressure, lung compliance, pathological changes, pulmonary vascular permeability, oxidative stress, inflammation, and apoptosis. Mice were infected with overexpression (OE)‐mitogen‐activated protein kinase kinase kinase 8 (MAP3K8) adenovirus and treated with DEX. MAP3K8 expression was examined in mouse lung tissue and pulmonary microvascular endothelial cells (PMVECs). Cells were infected using OE‐MAP3K8 lentivirus and treated with DEX, followed by detection of cell viability and apoptosis, VE‐cadherin and α‐E‐catenin, and pro‐inflammatory factors. Rescue experiments were performed by MAP3K8 overexpression and combined extracellular signal‐regulated protein kinase (ERK) pathway blocker, PD98059. The results demonstrated that DEX protected mice from LIRI. DEX inhibited MAP3K8 expression. MAP3K8 overexpression increased ERK1/2 phosphorylation and activated the ERK pathway. Upregulation of MAP3K8 impaired the protective effect of DEX in vivo and in vitro, which was reversed by the ERK inhibitor PD98059. Overall, DEX achieved its protective effect against LIRI by inhibiting the MAP3K8‐ERK axis.

## 1 Introduction

Lung transplantation is the most effective intervention for end‐stage lung disease, while its effectiveness is restricted by lung ischemia/reperfusion injury (LIRI) [1]. LIRI is marked by sterile inflammation, nonspecific alveolar damage, endothelial dysfunction, impaired oxygen exchange, and lung edema, resulting in notable morbidity and mortality [2]. Despite advancements in lung preservation, surgical management, and immunosuppressive therapy, approximately 54.3% of patients die within 5 years of transplantation [3]. Currently, there are no clinically available drugs to prevent IRI, and treatment strategies are limited to maintaining function [4]. Hence, it is crucial to illustrate the pathogenesis of LIRI and uncover new therapeutic methods.

Dexmedetomidine (DEX) is a highly selective α2 receptor agonist routinely utilized in the clinic for sedation and anesthesia, with protective effects against brain injury and IRI [5]. DEX remarkably mitigates apoptosis in the lungs and slightly improves oxygenation in mice after renal IRI; in vitro, DEX significantly improved viability and reduced apoptosis in pulmonary microvascular endothelial cells (PMVECs) [6]. Notably, in the hepatic IRI model, DEX alleviates histopathological injury and restores liver function by inhibiting the activation of c‐Jun NH2‐terminal protein kinase (JNK) and p38 mitogen‐activated protein kinase (MAPK) pathways, reducing blood–brain barrier permeability [7]. Our preliminary bioinformatics prediction identified mitogen‐activated protein kinase kinase kinase 8 (MAP3K8, also known as Tpl‐2) as a target of DEX, which piqued our attention that DEX might relieve LIRI by manipulating MAP3K8.

MAP3K8 is primarily expressed in the immune system, with important roles in epithelial inflammation [8]. MAP3K8 is upregulated after IRI in vivo and hypoxia/reoxygenation (H/R) injury in vitro [9]. MAPKs are serine–threonine protein kinases activated upon extracellular and intracellular stimuli, like oxidative stress, cytokines, and growth factors [10]. Three major MAPK signaling pathways, JNK, p38 MAPK, and extracellular signal‐regulated protein kinase1/2 (ERK1/2), regulate multiple inflammatory and related diseases [11]. MAPK activation by IRI in the heart and the liver has been reported [12, 13]. In addition, p38 MAPK inhibition lessens LIRI by maintaining endothelial barrier integrity and decreasing blood–air barrier hyperpermeability [14]. Various stimuli induce ERK1/2 phosphorylation, and abnormal activation of ERK1/2 signaling is strongly correlated with IRI [15]. Considering the significant involvement of MAPKs in LIRI, exploring how to obtain the optimal protective effects by MAPK inhibition requires exploration. As such, the roles of DEX and downstream MAP3K8 in LIRI were investigated in this study. It is hypothesized that DEX ameliorates LIRI via the MAP3K8/ERK1/2 axis. Understanding the role of DEX in LIRI is crucial for developing new therapeutic strategies.

## 2 Materials and Methods

### 2.1 LIRI Modeling in Mice

Male C57BL/6 mice (8 weeks old, 18–22 g) were purchased from Beijing Vital River Laboratory Animal Technology (Beijing, China). All mice used in this study were randomized. Mice were anesthetized by peritoneal injection of 200 mg/kg tribromoethanol and placed on a heating pad to maintain body temperature. Mice were subcutaneously injected with 0.1 mg/kg Buprenorphine before skin incision to minimize pain, distress, and discomfort. Following appropriate anesthesia, mice were intubated by tracheotomy and linked to a rodent ventilator (7025, Ugo Basile, Comerio, Varese, Italy) with a tidal volume of 10 μL/g and a respiratory rate set at 120 breaths/min. After thoracotomy, the left hilum of I/R mice was occluded with noninvasive microclamps and ischemia unilaterally for 1 h, followed by 2‐h reperfusion. In mice with one‐lung ventilation, the tidal volume was dropped to 6 μL/g, and the respiratory rate was enhanced to 150 breaths/min. Mice in the sham group received the same anesthesia and surgery, except for hilum occlusion [16].

DEX (purity: 99.93%, HY‐12719, MedChemExpress, Monmouth Junction, NJ, USA) was dissolved in sterile saline and injected intravenously at 25 μg/kg/h 1 h before ischemia. Control mice were administered intravenously with sterile saline [17]. PD98059 (10 μmol/L) (purity: 99.96%, HY‐12028, MedChemExpress) was applied by intraperitoneal injection 1 h before ischemia to inhibit the ERK pathway, and control mice were administered intraperitoneally with dimethylsulfoxide (DMSO) [18]. Overexpression (OE)‐MAP3K8 adenovirus and control adenovirus were injected via the tail vein 48 h before I/R. All adenoviruses were purchased from VectorBuilder (Guangzhou, Guangdong, China), and the adenovirus titer was 10^10^ PFU/mL [19]. After the experiment, mice were euthanized via intraperitoneal injection of 150 mg/kg sodium pentobarbital, and blood and lung samples were collected for further analyses. All animal experiments were ratified by the Ethics Committee for the Management of Laboratory Animals of the Second Affiliated Hospital of Mudanjiang Medical University and followed the guidelines for the use of laboratory animals.

### 2.2 Cell Culture

Mouse primary PMVECs (MIC‐iCell‐a001, iCell Bioscience, Shanghai, China) were cultured in gelatin‐coated endothelial cell complete medium (CM‐ZY001, Pricella, Houston, Texas, USA). For hypoxia exposure, 2 × 10^5^ PMVECs were seeded in 24‐well plates for 48 h, washed with phosphate‐buffered saline (PBS), added to serum/dextrose‐free endothelial cell complete medium, and then placed in a humidified and hypoxic chamber at 37°C. After 3 h of hypoxia, the chambers were opened, and the cells were incubated in a humidified incubator at 37°C with 5% CO_2_ for 2 h (reoxygenation). Normal cells received the same procedure as described above, except that hypoxic exposure was replaced by normoxic exposure in an endothelial cell complete medium for 5 h of normoxia [20]. PMVECs were cultured for 24 h and exposed to 0.1 μM DEX or control PBS, followed by H/R [6]. PMVECs were incubated with 20 μM PD98059 for 3 h or the same volume of DMSO for 3 h [21]. Before the H/R procedure, PMVECs were infected with OE‐MAP3K8 or control lentiviruses (with a viral titer of 10^8^ TU/mL) and further screened to obtain stably transfected cells using puromycin.

### 2.3 Pulmonary Function Measurement

After reperfusion, lung function was assessed with an experimental animal lung function testing system (Data Sciences International, St. Paul, Minnesota, USA). Mice were anesthetized by peritoneal injection of 200 mg/kg of tribromoethanol, and a tracheotomy was performed. The mice were ventilated with room air at 100 strokes/min, with a tidal volume of 7 μL/g and a positive end‐expiratory pressure of 2 cm H_2_O. Mice were bled by transecting the inferior vena cava. The pulmonary artery was cannulated via the right ventricle, and the left ventricle was cannulated and deflated via a small incision in the apex of the heart. Afterward, the lungs were perfused with Krebs‐Hensleit's solution (PB180348, Pricella) containing 2% albumin, 0.1% glucose, and HEPES free acid (60110ES60, Yeasen, Shanghai, China) (340 mOsm/kg H_2_O) at a constant flow rate of 60 μL/g/min. The perfusate buffer and isolated lungs were kept at 37°C in a circulating water bath. After correct perfusion and ventilation, the lungs were kept on the system for 5 min before data were recorded for 10 min, and hemodynamic and lung indexes were documented using the PULMODYN data acquisition system.

### 2.4 Histological Analysis

Lung tissues were washed with 30 mL of pre‐cooled PBS. PBS was injected into the right ventricle to eliminate residual blood. Then, lung samples were fixed in 4% paraformaldehyde for 24 h, embedded in paraffin, sliced, and placed on slides. After dewaxing and rehydration with different concentrations of xylene and ethanol, the sections were stained with hematoxylin (H3136, Merck, Darmstadt, Germany) for 7 min, rinsed with running water for 5 min, soaked in 1% acidic alcohol for 1 min, and counterstained with eosin Y solution (1.09844, Merck) for 2 min. Finally, the plates were dehydrated and sealed with neutral resin. A scoring system was adopted to assess pathologic injury to lung tissue: Pathological indicators included alveolar wall thickness (from 0 to 3), interstitial lung edema (from 0 to 2), lung hemorrhage (from 0 to 2), and inflammatory cell infiltration (from 0 to 3) [22].

### 2.5 Lung W/D Ratio

The lung W/D weight ratio was computed to check pulmonary edema. The left upper lobe was cleaned and weighed to get a wet weight, and then the lung was placed in an oven at 80°C for 12 h to get a dry weight and evaluate the W/D ratio.

### 2.6 Analysis of Protein Concentration in Bronchoalveolar Lavage Fluid (BALF)

After reperfusion, the neck skin of mice was incised, and the thyroid gland was separated from the trachea by blunt dissection. Surgical sutures were then performed behind the separated trachea. A small incision was made at the upper end of the trachea, and a catheter (0.7 mm in diameter) was quickly inserted, which was linked to a small animal indwelling needle (22 G). The catheter and trachea were fastened together with surgical sutures. After that, a 1 mL syringe was filled with saline and gently injected into the trachea, followed by aspiration of 0.5 mL of saline, which was repeated three times. Protein concentration in BALF was detected using a Bradford protein concentration assay kit (P0006, Beyotime, Shanghai, China). Finally, 5 μL of samples were added to the 96‐well plate, followed by 250 μL of G250 staining solution, and the absorbance at 595 nm was detected using a microplate reader.

### 2.7 Assessment of Lung Myeloperoxidase (MPO) Activity

An MPO test kit (A044‐1‐1, Nanjing Jiancheng Bioengineering Institute, Nanjing, Jiangsu, China) was used to assess MPO activity in mouse lung tissues. Mouse lung tissue was made into a 5% tissue homogenate at a weight‐to‐volume ratio of 1:19 with cold isotonic sodium chloride solution, followed by mixing with the assay reagent. The absorbance at 460 nm was tested using a spectrophotometer immediately after removal from the water bath at 60°C for 10 min.

### 2.8 Measurement of Reduced Glutathione (GSH), Oxidized Glutathione (GSSG), and GSH/GSSG

The T‐GSH/GSSG Colorimetric Assay Kit (E‐BC‐K097‐S, Elabscience Biotechnology Co. Ltd., Wuhan, Hubei, China) was used. A glass homogenizer was used to homogenize mouse lung tissues at 4°C after the addition of protein remover according to the manufacturer's instructions and centrifuged for 10 min. The samples were incubated with the reaction working solution for 5 min and then with the substrate solution for 25 min (both at room temperature). The reaction termination solution was added, and the absorbance was measured at 412 nm using a spectrophotometer, at which time the GSH content was obtained. For GSSG content, GSH scavenging auxiliary solution and GSH scavenger were added to the supernatant samples separated from the tissue homogenate after adding protein remover. After 1 h of reaction, the samples were incubated with the reaction working solution for 5 min and with the substrate for 25 min (all at room temperature). Finally, the reaction termination solution was added, and the absorbance at 412 nm was measured under the spectrophotometer, and GSSG content was calculated.

### 2.9 Enzyme‐Linked Immunosorbent Assay (ELISA)

Blood samples were collected from the right femoral artery by catheter insertion and coagulated at room temperature, and rapidly centrifuged at 1000 *g* for 5 min to harvest the supernatant. Interleukin 1β (IL‐1β, GEM0002‐48T, Servicebio, Wuhan, Hubei, China), interleukin 6 (IL‐6, GEM0001‐48T, Servicebio), and tumor necrosis factor α (TNF‐α, GEM0004‐48T, Servicebio) were measured using ELISA kits. Samples and antibodies were added to the plates, which were sealed, shaken, and incubated for 2 h. After washing, the plate was drained upside down on absorbent paper, and the liquid in the wells was discarded. After the addition of SA‐HRP, the plate was sealed, shaken, and incubated for 30 min. Then TMB substrate solution was put into each well, followed by plate sealing and development away from light. Finally, the termination solution was added, and absorbance at 450 nm was read after ensuring no water droplets at the bottom and no air bubbles in the wells.

### 2.10 Reverse Transcription‐Quantitative Polymerase Chain Reaction (RT‐qPCR)

Total RNA extraction from lung tissues and cells was conducted by TRIzol reagent (15596018CN, Thermo Fisher, Waltham, MA, USA), followed by reverse transcription to cDNA using PrimeScrip RT reagent kit (Perfect Real Time) (RR037Q, TaKaRa, Dalian, Liaoning, China). SYBR Green PCR premix (4344463, Thermo Fisher) and StepOne Real‐Time Fluorescence qPCR System (4376357, Thermo Fisher) were utilized for real‐time PCR. The relative expression of genes was computed utilizing the 2^−ΔΔCt^ method with GAPDH as a control. PCR primers used are listed in Table 1.

**TABLE 1:** Primer sequences for RT‐qPCR.

Gene	Forward sequence (5′–3′)	Reverse sequence (5′–3′)
MAP3K8 (mouse)	CTTTGAACGGAAGAGGCTGCTG	GAACGCTGTCTCCTGAGCACTT
IL‐1β (mouse)	TGGACCTTCCAGGATGAGGACA	GTTCATCTCGGAGCCTGTAGTG
IL‐6 (mouse)	TACCACTTCACAAGTCGGAGGC	CTGCAAGTGCATCATCGTTGTTC
TNF‐α (mouse)	GGTGCCTATGTCTCAGCCTCTT	GCCATAGAACTGATGAGAGGGAG
GAPDH (mouse)	CATCACTGCCACCCAGAAGACTG	ATGCCAGTGAGCTTCCCGTTCAG

Abbreviations: GAPDH, glyceraldehyde‐3‐phosphate dehydrogenase; IL‐1β, interleukin 1β; IL‐6, interleukin 6; MAP3K8, p38 mitogen‐activated protein kinase; TNF‐α, tumor necrosis factor α.

### 2.11 Immunohistochemistry (IHC)

IHC assays were performed on lung tissue samples isolated from mice. Mouse lung tissues were fixed with formalin, embedded in paraffin, and sectioned at 4‐μm, which were then deparaffinized with xylene and graded alcohol, followed by heat‐induced retrieval in citrate buffer. Next, sections were sealed with 5% goat serum and probed overnight with a primary antibody anti‐MAP3K8 (1:100, orb670747, Biorbyt, Waterbeach, Cambridge, UK) and with a secondary antibody goat anti‐rabbit IgG H&L (HRP) (1:1000, ab6721, Abcam, Cambridge, MA, USA). DAB (36201ES03, Yeasen) was added for color development, and hematoxylin for counterstaining. Finally, microscopic imaging analysis was performed, and the results were manifested as the percentage of positive cell staining.

### 2.12 Western Blot (WB)

Total protein in the cells was extracted with RIPA lysis buffer (20‐188, Merck) and determined using the BCA kit (71285‐M, Merck). An equal amount of protein was separated via electrophoresis and transferred to PVDF membranes (WJ002S, Epizyme Biotech, Shanghai, China). The membranes were then enclosed in 5% goat serum and probed at 4°C with primary antibodies anti‐MAP3K8 (1:1000, orb1259419, Biorbyt), anti‐Cleaved‐Caspase‐3 (1:1000, PA5‐114687, Thermo Fisher), anti‐VE‐cadherin (1:1000, ab33168, Abcam), anti‐α‐E‐catenin (1:1000, 12831‐1‐AP, Proteintech, Wuhan, Hubei, China), anti‐p‐ERK1/2 (1:1000, 44‐680G, Thermo Fisher), anti‐ERK1/2 (1:10000, ab184699, Abcam), and anti‐GAPDH (1:2500, ab9485, Abcam) overnight. After three washes in TBST, the membranes were probed with the secondary antibody goat anti‐rabbit IgG H&L (HRP) (1:2000, ab6721, Abcam). Bands were exposed using the Novex ECL Chemiluminescent Substrate Kit (WP20005, Thermo Fisher) and quantified by densitometric analysis using Image J software.

### 2.13 Cell Viability Assay

Cell viability assay was carried out using the CCK‐8 kit (40203ES60, Yeasen). Cell suspensions were seeded in 96‐well plates (37°C, 5% CO_2_) for 24 h. Then, each well was added with 10 μL of CCK‐8 solution for 2 h. The absorbance was read at 450 nm.

### 2.14 TUNEL Assay

Apoptosis was detected using a one‐step TUNEL Apoptosis Assay Kit (C1086, Beyotime). Cells were fixed for 30 min with 4% paraformaldehyde and incubated with immunostaining permeabilizing buffer (BL935B, Biosharp, Hefei, Anhui, China) for 5 min. Mouse lung tissue paraffin sections were first deparaffinized with xylene, hydrated with a gradient of ethanol, and reacted with proteinase K solution (P1120, Solarbio, Beijing, China) at 37°C for 30 min. Cells and tissue sections were incubated with TUNEL assay solution at 37°C in the dark for 60 min. The samples were stained with DAPI, blocked with an anti‐fluorescence quenching solution, and examined under a fluorescence microscope.

### 2.15 Immunofluorescence Staining

The treated mouse PMVECs were fixed with 4% paraformaldehyde, permeabilized with 0.5% Triton‐X‐100 (HY‐Y1883A, MedChemExpress) and sealed with 3% goat serum for 1 h, followed by overnight incubation with primary antibodies anti‐VE‐cadherin (1:1000 ab33168, Abcam) and anti‐α‐E‐catenin (1:100, 12831‐1‐AP, Proteintech) at 4°C. The cells were then probed with secondary antibody goat anti‐rabbit IgG H&L (1:1000, ab150077, Abcam) for 2 h at 37°C and counterstained with DAPI solution (C0065, Solarbio). Finally, cell images were captured on a fluorescence microscope, and fluorescence intensity was analyzed using Image J.

### 2.16 Statistical Analysis

All experiments were repeated independently at least three times. Data were analyzed with Prism 8.0.2 (GraphPad Software Inc., San Diego, CA, USA). Data are presented as mean ± standard error of the mean. Pairwise comparisons of a single factor were analyzed by *t*‐test, while multigroup comparisons of a single factor were done by ANOVA, with the Tukey test for post hoc tests. *p* < 0.05 was deemed statistically significant.

## 3 Results

### 3.1 DEX Protects the Lungs From LIRI

**FIGURE 1:**
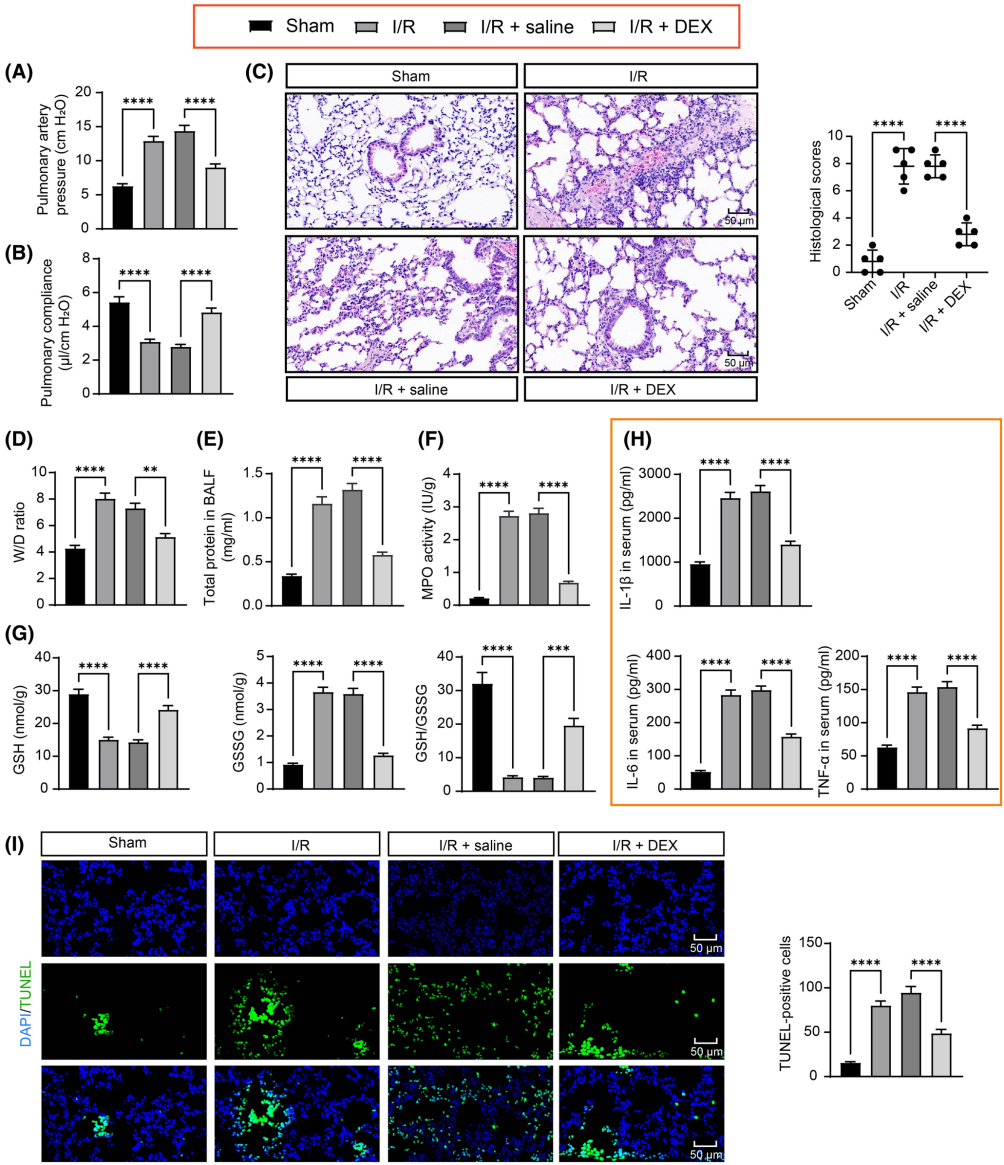
Protective effect of DEX on LIRI. (A) Detection of pulmonary arterial pressure in mice (*n* = 5); (B) detection of lung compliance in mice (*n* = 5); (C) HE analysis of LIRI in mice and histological scoring (*n* = 5); (D) detection of wet/dry weight ratio of lung tissues (*n* = 5); (E) detection of BALF protein concentration in lung tissues (*n* = 5); (F) detection of lung MPO activity (*n* = 5); (G) detection of GSH, GSSG, and GSH/GSSG in mouse lung tissues (*n* = 5); (H) ELISA to detect the concentrations of IL‐1β, IL‐6, and TNF‐α in serum of mice (*n* = 5); (I) TUNEL to detect apoptosis in mouse lung tissues (*n* = 5). Multigroup comparisons were made by ANOVA (ABCDEFGHI), **p < 0.01, ***p < 0.001, ****p < 0.0001, versus sham group or I/R + saline.

Lung function measurements revealed that LIRI significantly increased pulmonary arterial pressure and reduced lung compliance, and mice pre‐treated with DEX showed significant improvement in lung function (Figure 1A,B). Histological analysis revealed that LIRI resulted in interstitial edema, pulmonary capillary siltation, inflammatory cell infiltration, and alveolar wall thickening, with a significant increase in histological scores. DEX‐treated mice showed significant improvement in pulmonary edema and lung histopathological damage, with a significant decrease in histological scores (Figure 1C). The W/D ratio and BALF protein concentration were upregulated in the I/R group compared to the sham group and significantly downregulated after DEX treatment (Figure 1D,E). MPO activity, a marker related to oxidative stress, was increased in I/R‐stimulated lungs but suppressed by DEX treatment (Figure 1F). The activity of GSH was reduced, and GSSG was elevated, which resulted in a lower GSH/GSSG ratio in lung tissues of I/R mice, and these markers were reversed by DEX treatment (Figure 1G). ELISA revealed that the serum concentrations of IL‐1β, IL‐6, and TNF‐α were elevated in I/R mice, and DEX treatment decreased the concentrations of these factors (Figure 1H). TUNEL assay manifested that the apoptosis levels of lung tissue were upregulated in I/R mice, and DEX treatment significantly reduced apoptosis (Figure 1I). In conclusion, DEX protects the lungs from LIRI.

### 3.2 DEX Targets and Inhibits MAP3K8 Expression

**FIGURE 2:**
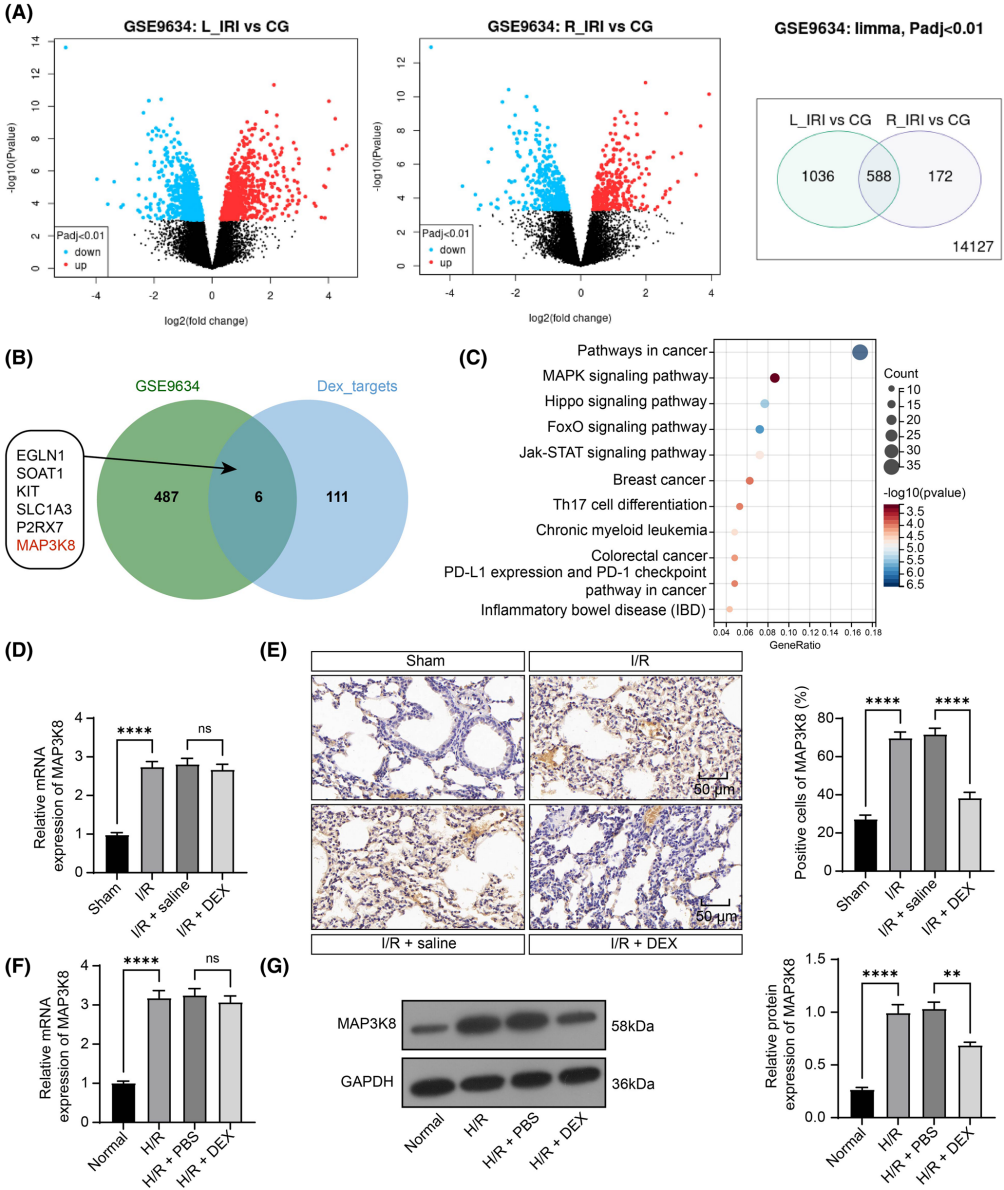
DEX has inhibitory effects on MAP3K8 expression. (A) Gene expression profiles of RNA‐seq data were downloaded from the GSE9634 database and screened to obtain DEGs with adj. *p* < 0.01 as the threshold (CG was the control group, L‐IRI was the left lung of the experimental group, and R‐IRI was the right lung of the experimental group); (B) the intersection of target proteins of DEX downloaded from Super‐PRED and the obtained DEGs from the GSE9634 database; (C) DEGs in the GSE9634 dataset were subjected to KEGG enrichment analysis in SangerBOX; (D) RT‐qPCR detected MAP3K8 expression in lung tissues (*n* = 5); (E) IHC detected MAP3K8 expression in lung tissues (*n* = 5); (F) RT‐qPCR detected MAP3K8 expression in PMVECs (*n* = 5); (G) WB detection of protein expression of MAP3K8 in PMVECs (*n* = 3). Multigroup comparisons were done by ANOVA (DEFG). Ns > 0.05, **p < 0.01, ****p < 0.0001, versus sham group, normal group, I/R + saline group, or H/R + PBS group.

Gene expression profiles of RNA‐seq data from the left lung of Norway rats under in situ warm ischemic conditions versus normal lungs were downloaded from the GSE9634 dataset in the GEO database and screened to obtain differentially expressed genes (DEGs) with a threshold value of adj. *p* < 0.01 to obtain 588 DEGs, which contained null genes, and 493 genes were obtained after deletion of null genes (Figure 2A). The direct targets of DEX were predicted in the Super‐PRED database (https://prediction.charite.de/index.php). The UniProt Accession of DEX direct targets was transformed into GeneSymbol, after which they were intersected with the obtained DEGs in Jvenn (https://jvenn.toulouse.inrae.fr/app/example.html). Six intersecting genes, EGLN1, SOAT1, KIT, SLC1A3, P2RX7, and MAP3K8, were obtained (Figure 2B). KEGG enrichment analysis (https://www.kegg.jp/kegg/pathway.html) was conducted using DEGs (Figure 2C) at SangerBOX (http://vip.sangerbox.com). Even though the Pathways in cancer was the most enriched pathway according to *p*‐value, we exclude it due to the limited relevance to this study. Therefore, we focused on the second most differentially expressed enriched MAPK signaling pathway. Among the six intersecting genes, KIT and MAP3K8 were both enriched in the MAPK signaling pathway. However, KIT (LogFC = −1.243) was under‐expressed in the GSE9634 dataset, and thus it may not be primarily responsible for proinflammatory responses in the disease. MAP3K8 (LogFC = 2.225) was enhanced in the lung tissues of I/R mice. Therefore, MAP3K8 was chosen as the drug target.

RT‐qPCR and IHC assays uncovered that MAP3K8 levels were upregulated in the lung tissues of I/R mice. The mRNA expression of MAP3K8 showed no significant change after pre‐administration of DEX, while the protein expression was significantly downregulated (Figure 2D,E). Before H/R exposure, PMVECs were treated with 0.1 μM DEX, and RT‐qPCR and WB analyses revealed that MAP3K8 was notably upregulated in PMVECs exposed to the H/R group relative to cells under normoxic conditions. By contrast, the mRNA expression of MAP3K8 was not notably changed, while the protein level was significantly downregulated in cells pre‐treated with DEX (Figure 2F,G). In summary, DEX inhibits MAP3K8 protein expression.

### 3.3 DEX Ameliorates H/R‐Induced Injury in PMVECs by Downregulating MAP3K8

**FIGURE 3:**
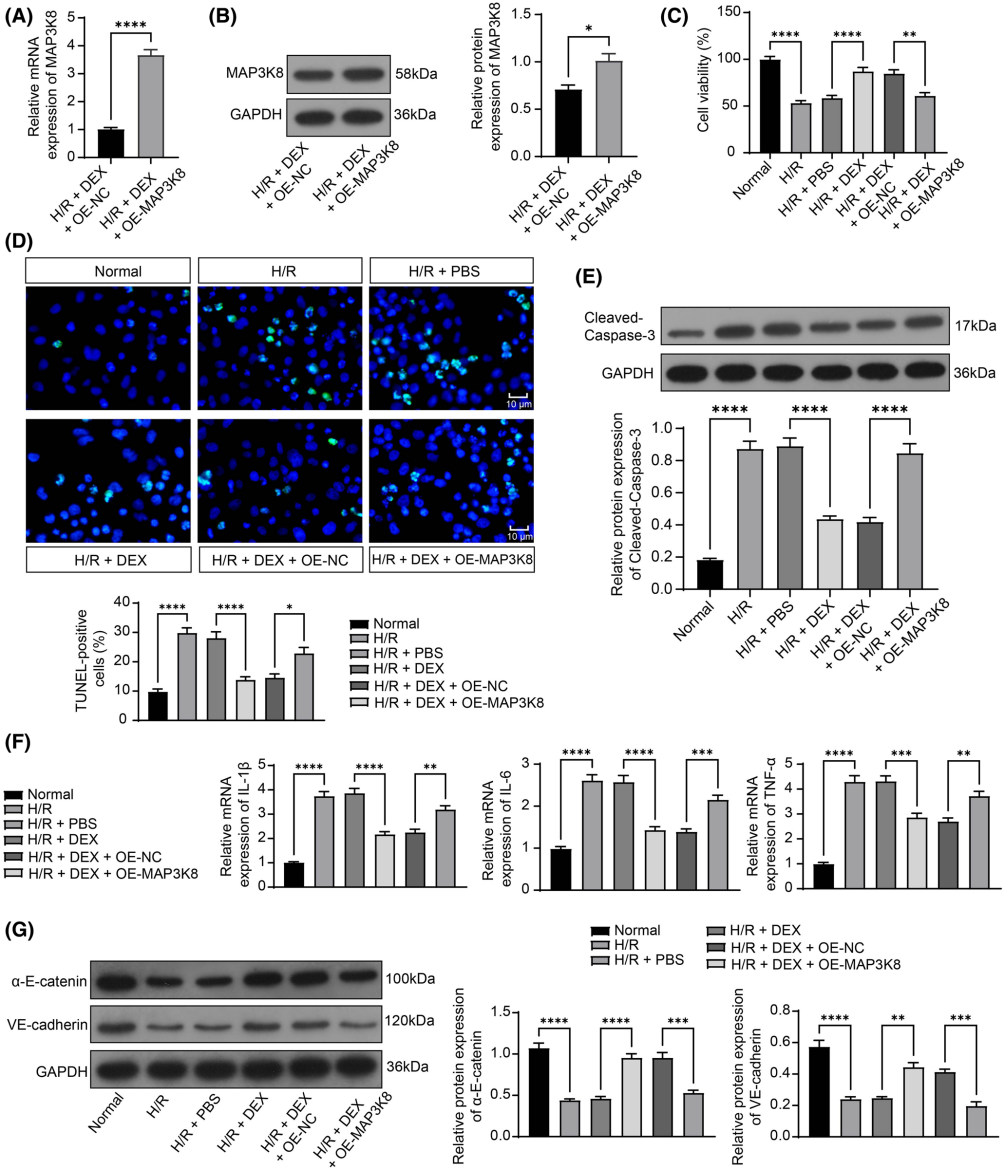
DEX ameliorates H/R‐induced injury in vitro by inhibiting MAP3K8. (A) RT‐qPCR to assess MAP3K8 expression in PMVECs infected with lent viral vector overexpressing MAP3K8 (*n* = 5); (B) WB to detect MAP3K8 level in PMVECs (*n* = 3); (C) CCK‐8 to detect PMVEC viability (*n* = 5); (D) TUNEL assay to examine PMVEC apoptosis (*n* = 5); (E) WB detected Cleaved‐Caspase‐3 in PMVECs (*n* = 3); (F) RT‐qPCR to assess IL‐1β, IL‐6, and TNF‐α expression in PMVECs (*n* = 5); (G) WB detection of VE‐cadherin and α‐E‐catenin protein expression in PMVECs (*n* = 3). Multigroup comparisons were done by unpaired *t*‐test (AB) or ANOVA (CDEFG). *p < 0.05, **p < 0.01, ***p < 0.001, ****p < 0.0001, versus the normal group, H/R + PBS group, or H/R + DEX + OE‐NC group.

PMVECs were infected with OE‐MAP3K8 lentivirus, and the overexpression efficiency was confirmed by RT‐qPCR (Figure 3A). WB assay elicited that MAP3K8 overexpression reversed the repressing effect of DEX on MAP3K8 protein expression (Figure 3B). CCK‐8 assay found that DEX treatment increased cell viability even after H/R exposure, while the overexpression of MAP3K8 notably downregulated cell viability (Figure 3C). TUNEL assay unraveled that apoptosis was upregulated in the H/R group, downregulated in the H/R + DEX group, and greatly upregulated in the H/R + DEX + OE‐MAP3K8 group (Figure 3D). WB analysis noted that H/R exposure upregulated Cleaved‐Caspase‐3 in PMVECs, which was averted by MAP3K8 overexpression (Figure 3E). IL‐1β, IL‐6, and TNF‐α were greatly elevated in the H/R group, downregulated in the I/R + DEX group, and upregulated in the H/R + DEX + OE‐MAP3K8 group (Figure 3F). WB analysis revealed that H/R treatment reduced VE‐cadherin and α‐E‐catenin in PMVECs, and MAP3K8 overexpression reversed these protein levels in the presence of DEX treatment (Figure 3G).

### 3.4 Overexpression of MAP3K8 Impairs the Therapeutic Effect of DEX on LIRI in Mice

**FIGURE 4:**
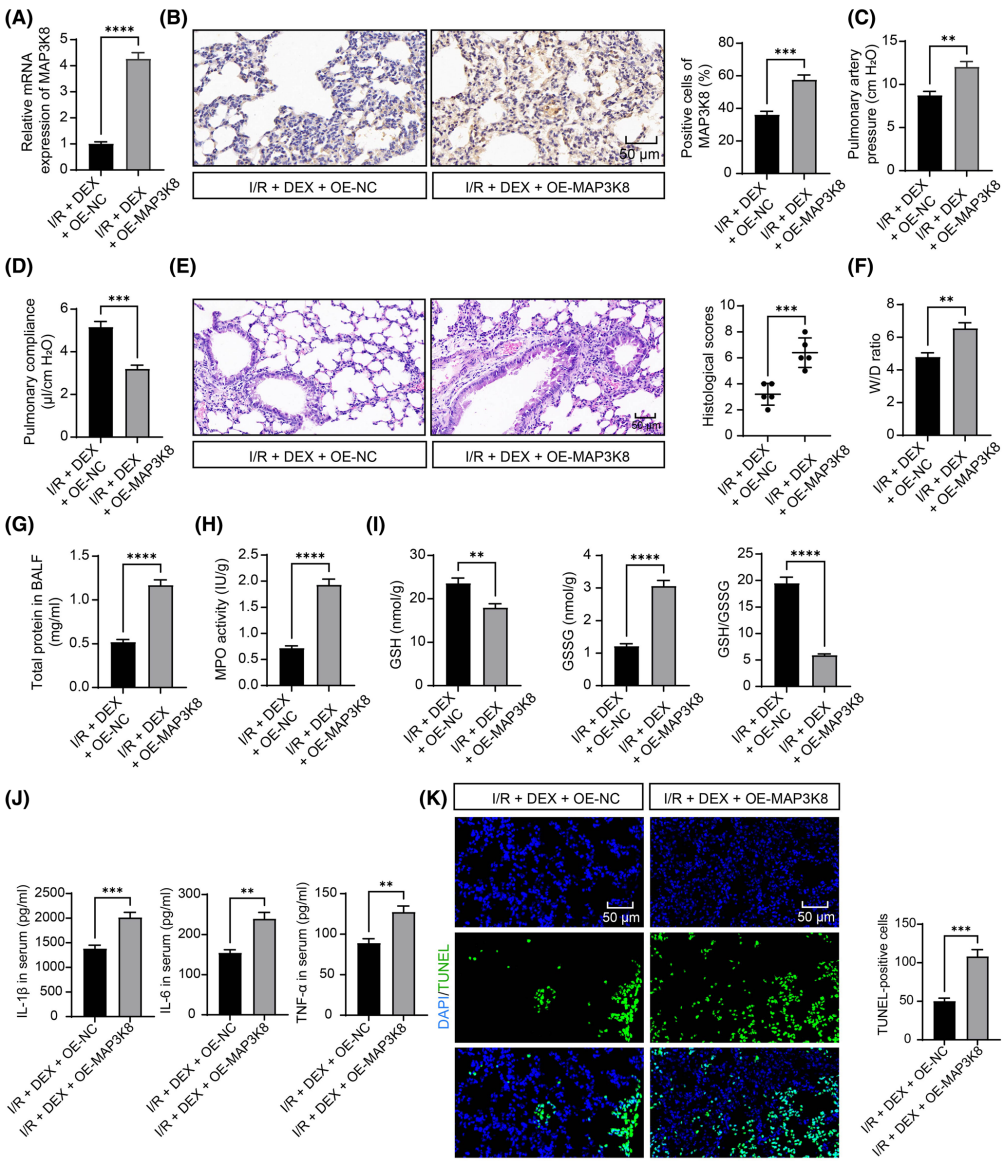
DEX downregulates MAP3K8 to alleviate LIRI in mice. (A) RT‐qPCR detected PAM3K8 expression in lung tissues of mice after MAP3K8 overexpression (*n* = 5); (B) IHC to detect MAP3K8 expression in lung tissues (*n* = 5); (C) detection of pulmonary arterial pressure in I/R mice (*n* = 5); (D) detection of lung compliance in I/R mice (*n* = 5); (E) HE analysis and histological scoring of LIRI in I/R mice after MAP3K8 overexpression (*n* = 5); (F) detection of W/D ratio in lung tissues (*n* = 5); (G) detection of BALF protein content in lung tissues (*n* = 5); (H) detection of MPO activity in lungs (*n* = 5); (I) detection of GSH, GSSG, and GSH/GSSG in lung tissues (*n* = 5); (J) ELISA for IL‐1β, IL‐6, and TNF‐α in serum (*n* = 5); (K) TUNEL assay to analyze the apoptosis levels of lung tissue (*n* = 5). Pairwise comparisons were done using the unpaired *t*‐test (ABCDEFGHIJK). **p < 0.01, ***p < 0.001, ****p < 0.0001, versus the I/R + DEX + OE‐NC group.

OE‐MAP3K8 adenovirus was injected into mice 48 h before I/R, and RT‐qPCR and IHC verified the interference effect of adenovirus on MAP3K8 (Figure 4A,B). Lung function measurements revealed that mice overexpressing MAP3K8 had significantly deteriorated lung function, increased pulmonary artery pressure, and decreased lung compliance (Figure 4C,D). Histological analysis revealed that mice treated with OE‐MAP3K8 had visibly higher histologic scores (Figure 4E). Overexpression of MAP3K8 notably upregulated lung W/D ratio and BALF protein concentration (Figure 4F,G) and greatly increased MPO activity (Figure 4H). Decreased GSH activity and increased GSSG led to a reduced GSH/GSSG ratio in the lung tissues in response to enforced expression of MAP3K8 (Figure 4I). ELISA disclosed that serum IL‐1β, IL‐6, and TNF‐α levels were notably increased in the I/R + DEX + OE‐MAP3K8 group (Figure 4J). TUNEL analysis revealed that apoptosis levels of lung tissue were greatly enhanced after OE‐MAP3K8 (Figure 4K).

### 3.5 DEX Ameliorates H/R‐Induced Injury in PMVECs by Inhibiting MAP3K8 and Blocking the ERK Signaling

**FIGURE 5:**
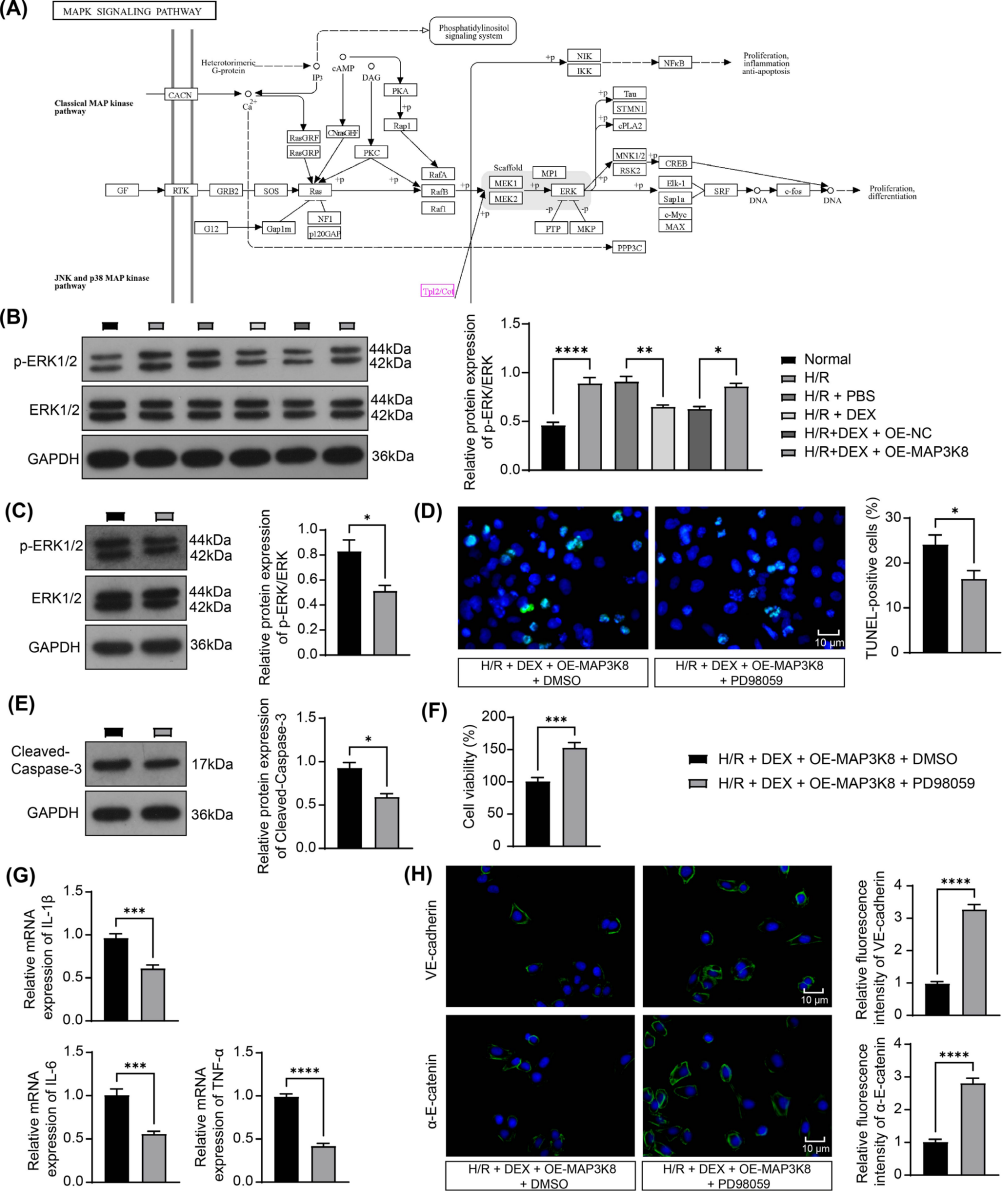
DEX ameliorates H/R‐induced injury in PMVECs via the MAP3K8‐ERK axis. (A) KEGG enrichment of MAP3K8‐mediated ERK pathway; (B) WB detection of ERK phosphorylation in PMVECs (*n* = 3); (C) WB detection of ERK phosphorylation in MAP3K8‐overexpressing PMVECs after PD98059 treatment (*n* = 3); (D) TUNEL assay to detect apoptotic PMVECs (*n* = 5); (E) WB detection of Cleaved‐Caspase‐3 in PMVECs (*n* = 3); (F) CCK‐8 detected PMVEC viability (*n* = 5); (G) RT‐qPCR detected IL‐1β, IL‐6, and TNF‐α in PMVECs (*n* = 5); (H) immunofluorescence detected VE‐cadherin and α‐E catenin expression in PMVECs (*n* = 5). Multigroup comparisons were analyzed by ANOVA (B), and pairwise comparisons were done by unpaired *t*‐test (CDEFGH). *p < 0.05, **p < 0.01, ***p < 0.001, ****p < 0.0001, versus H/R + DEX + OE‐MAP3K8 + DMSO group.

MAP3K8 was upstream of the ERK pathway in KEGG (Figure 5A). PMVECs were infected using OE‐MAP3K8 lentivirus. WB assay revealed that MAP3K8 overexpression increased the phosphorylated form of ERK1/2 (Figure 5B). PD98059 is an inhibitor of the ERK1/2 pathway, and PMVECs were treated with 20 μM PD98059 or control DMSO. WB assay revealed a significant downregulation of the phosphorylated form of ERK1/2 after PD98059 treatment (Figure 5C). TUNEL and WB analyses revealed that the apoptosis of PMVECs and Cleaved‐Caspase‐3 protein levels were notably reduced after the ERK signaling was blocked (Figure 5D,E), while cell viability was greatly enhanced (Figure 5F). RT‐qPCR revealed that pro‐inflammatory factors were significantly downregulated in the PMVECs with ERK signaling impairment (Figure 5G). Immunofluorescence detection revealed that VE‐cadherin and α‐E‐catenin labeling were greatly elevated after PD98059 treatment (Figure 5H).

### 3.6 DEX Protects Against LIRI in Mice by Blocking ERK Signaling Through Inhibition of MAP3K8

**FIGURE 6:**
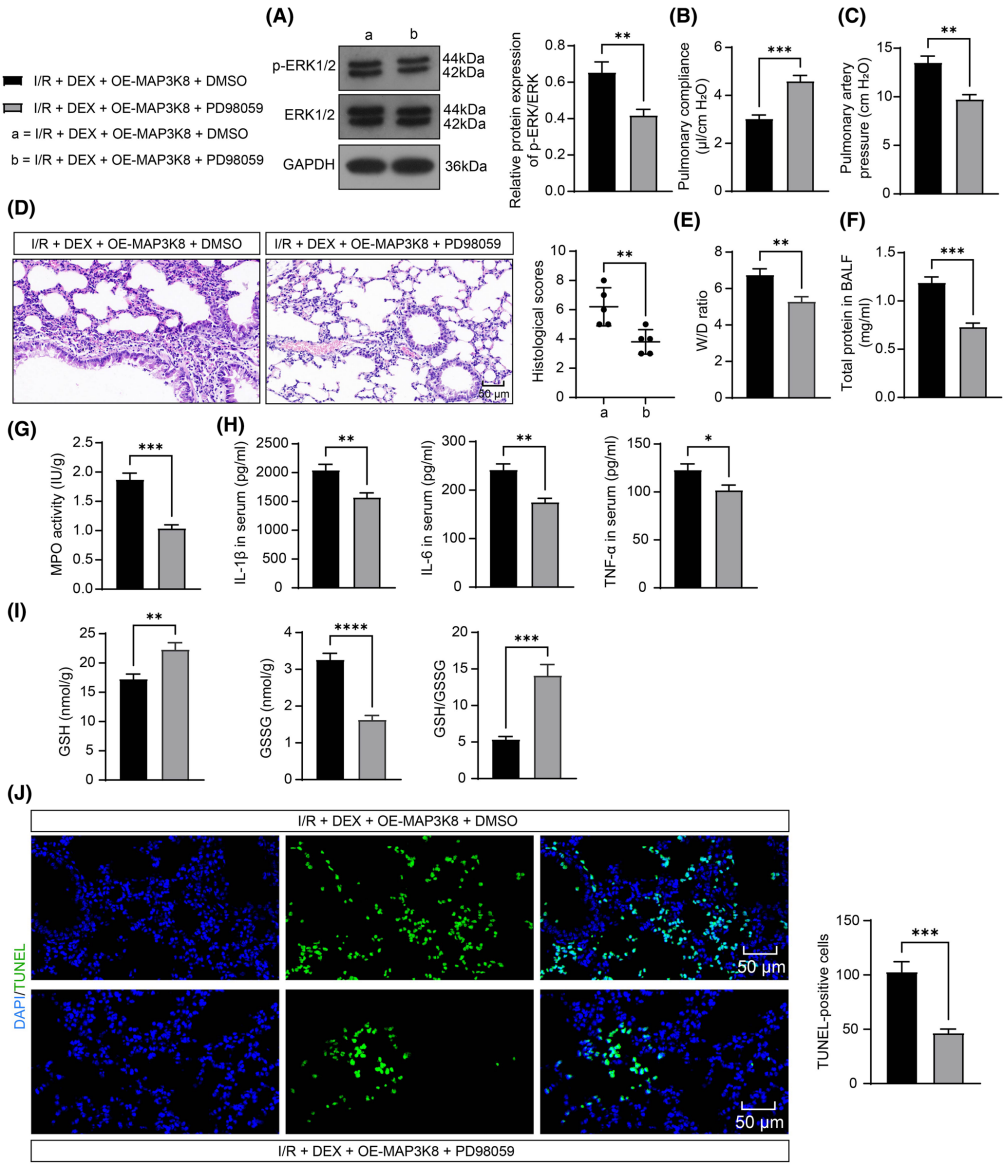
DEX inhibits the MAP3K8/ERK1/2 axis to achieve protection against LIRI in mice. (A) WB detection of p‐ERK1/2 protein expression in mouse lung tissues after pre‐treatment of PD98059 or DMSO and combined OE‐MAP3K8 (*n* = 5); (B) detection of lung compliance (*n* = 5); (C) detection of pulmonary artery pressure (*n* = 5); (D) HE analysis for IRI in mouse lungs and histological scoring (*n* = 5); (E) detection of W/D ratio in mouse lungs (*n* = 5); (F) detection of BALF protein in mouse lung tissue (*n* = 5); (G) detection of MPO activity in mouse lungs (*n* = 5); (H) ELISA for IL‐1β, IL‐6, and TNF‐α in mouse serum (*n* = 5); (I) detection of GSH, GSSG and GSH/GSSG in mouse lung tissues (*n* = 5); (J) TUNEL for apoptosis in mouse lung tissues (*n* = 5). Pairwise comparisons were analyzed by an unpaired *t*‐test (ABCDEFGHIJ). *p < 0.05, **p < 0.01, ***p < 0.001, ****p < 0.0001, versus I/R + DEX + OE‐MAP3K8 + DMSO group.

ERK signaling was blocked by intraperitoneal injection of PD98059 1 h before ischemia. In the I/R + DEX + OE‐MAP3K8 + PD98059 group, the protein expression of p‐ERK1/2 was notably diminished in lung tissues of mice (Figure 6A), along with increased lung compliance (Figure 6B) and reduced pulmonary arterial pressure (Figure 6C). Moreover, interstitial edema and inflammatory cell infiltration improved following PD98059 treatment, as evidenced by decreased histological scores (Figure 6D) and lowered lung W/D ratio and BALF protein concentration (Figure 6E,F). Furthermore, PD98059 treatment resulted in decreased MPO activity (Figure 6G), lowered IL‐1β, IL‐6, and TNF‐α (Figure 6H), enhanced GSH/GSSG (Figure 6I), and reduced apoptosis rate (Figure 6J).

## 4 Discussion

**FIGURE 7:**
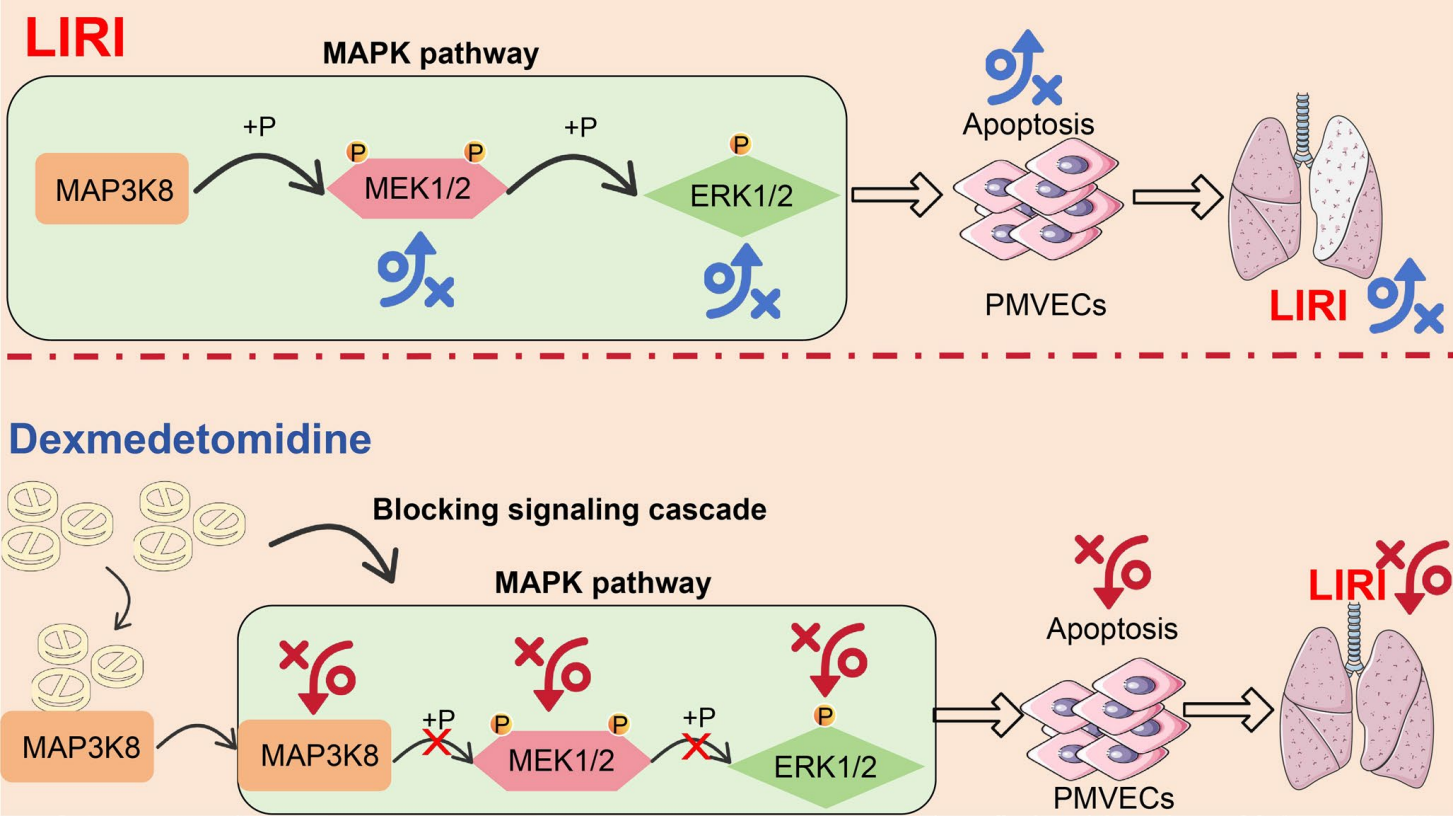
Schematic model for protective effects of DEX against LIRI in mice. DEX mitigates LIRI by blocking ERK1/2 phosphorylation and ERK signaling pathway activation through inhibition of MAP3K8.

Although lung transplant rates continue to rise every year, lung transplant outcomes are among the worst of all solid organ transplants due to IRI, with approximately 67% of patients dying within 10 years [23]. Compelling evidence has noted that DEX can protect IRI in multiple organs such as the intestine, kidney, myocardium, brain, lung, and liver through antioxidant, anti‐inflammatory, and anti‐apoptotic mechanisms [24]. We hypothesize that DEX achieves its protective effect in LIRI by inhibiting MAP3K8 to block the ERK pathway and thereby inhibit PMVEC dysfunction (Figure 7).

Mice displayed pulmonary dysfunction after I/R, as manifested by elevated airway resistance, pulmonary artery pressure, vascular permeability, proinflammatory cytokines in BALF, neutrophil infiltration, elevated MPO levels, pulmonary edema, and decreased pulmonary compliance [25]. Notably, our experimental data illustrated that DEX treatment improved lung function and reduced pulmonary edema and permeability, MPO activity, oxidative stress, inflammation, and apoptosis, showing significant protection against LIRI. GSH has vital functions involving antioxidant protection, redox homeostasis, DNA synthesis, and cell apoptosis [26]. GSH contents and GSH/GSSG ratio are diminished in oxidative stress conditions and may cause enhanced oxidative toxicity [27]. Lung W/D ratio and BALF protein concentration are common indices of pulmonary vascular permeability and typical traits of acute lung injury [16]. Endothelial cells in patients with systemic inflammation are often extensively damaged, especially in the microcirculatory system, possibly due to oxidative stress associated with tissue I/R [28]. Therefore, we focused on PMVECs in vitro. Endothelial junctions provide blood and lymphatic vessel integrity and control permeability, where VE‐cadherin and α‐E‐catenin are important [29]. Our results revealed that DEX treatment significantly increased the viability of H/R‐exposed PMVECs, reduced apoptosis, inhibited Cleaved‐Caspase‐3 protein expression, limited IL‐1β, IL‐6, and TNF‐α, and upregulated VE‐cadherin and α‐E‐catenin. Consistently, previous reports demonstrated that DEX significantly mitigated pathohistological lung injury and apoptosis in mice after I/R and inhibited loss of viability and apoptosis in PMVECs, with downregulated levels of caspase 3 and Bax in PMVECs [6]. DEX could notably reduce cerebral infarction in I/R rats, maintain mitochondrial functions, and enhance GSH levels [30]. DEX diminished serum inflammatory cytokine concentrations and oxidative stress, thus alleviating I/R‐associated acute kidney injury [31].

During the downstream investigation, we focused on MAP3K8 among various DEGs. Additionally, upregulated MAP3K8 was observed in lung tissue of I/R mice and H/R‐induced PMVECs, but downregulated after DEX treatment, without any change in the mRNA expression. MAP3K8 was significantly increased in spinal cord IRI [32]. MAP3K8 was upregulated after IRI in vivo and H/R injury in vitro, and MAP3K8 inhibition was associated with reduced infarct size and repressed cardiomyocyte pyroptosis and oxidative stress [9]. Our results also substantiated that overexpression of MAP3K8 impaired the therapeutic effect of DEX on LIRI. MAP3K8 positively regulates the MEK1/2 and ERK1/2 pathways to initiate an array of inflammatory responses [33]. DEX attenuates hepatic IRI‐induced apoptosis and oxidative stress by reducing ERK phosphorylation and blocking H/R‐induced MAPK signaling activation [7]. MAPKs were activated during simulated reperfusion, and silencing MAPKs attenuated oxidative stress, inflammation, and apoptosis in rat PMVECs [34]. DEX pretreatment considerably repressed neutrophil infiltration, pulmonary edema, and proinflammatory cytokines by decreasing phosphorylation of ERK1/2 [35]. Interestingly, MAP3K8 overexpression increased ERK1/2 phosphorylation in PMVECs in the present study. Furthermore, ERK1/2 inhibition through PD98059 treatment mitigated the effect of OE‐MAP3K8 on LIRI in vitro and in vivo. ERK is increased in IR‐induced ALI, and decreased ERK was associated with attenuated pulmonary hyperpermeability, neutrophilic infiltration, pulmonary edema, and proinflammatory cytokines [36]. Hypoxia increased the p‐ERK1/2 to ERK1/2 ratio in VECs, while PD98059 increased cell viability and alleviated the damage to cell morphology [37]. Septic serum promoted ERK1/2 phosphorylation in human umbilical vein endothelial cells, while pre‐treatment with the ERK1/2 inhibitor PD98059 decreased the septic serum‐induced inflammatory injury [38].

Our study also has certain limitations. First, molecular docking is needed to verify the physical interaction between DEX and the MAP3K8 domains. Second, DEX has been reported to protect against lung injury induced by limb I/R via the NF‐κB pathway [39], while MAP3K8 has been identified as the essential kinase that propels both MAPK and NF‐κB cascades [40]. We look forward to corroborating other mechanisms of cell dysfunction by MAP3K8 in the future. Third, investigating the synergistic effects of DEX with other pharmacological agents that target different pathways involved in I/R injury could enhance its protective effects. For instance, combining DEX with antioxidants or anti‐inflammatory drugs might provide a more comprehensive approach to mitigating lung I/R injury.

## 5 Conclusion

In summary, DEX inhibits PMVEC dysfunction and achieves its protective effect in LIRI by inhibiting MAP3K8 protein levels and blocking the ERK1/2 pathway. More research and clinical trials on DEX in the field of I/R injury are required, and other mechanisms involved in the protective effects of DEX also deserve attention.

## Conflicts of Interest

The authors declare no conflicts of interest.

## Data Availability

The data that support the findings of this study are available on request from the corresponding author. The data are not publicly available due to privacy or ethical restrictions.

## References

[1] M. Capuzzimati , O. Hough , and M. Liu , “Cell Death and Ischemia‐Reperfusion Injury in Lung Transplantation,” Journal of Heart and Lung Transplantation 41, no. 8 (2022): 1003–1013. 10.1016/j.healun.2022.05.013 35710485

[2] T. Talaie , L. DiChiacchio , N. K. Prasad , et al., “Ischemia‐Reperfusion Injury in the Transplanted Lung: A Literature Review,” Transplantation Direct 7, no. 2 (2021): e652. 33437867 10.1097/TXD.0000000000001104 PMC7793349

[3] M. Valapour , C. J. Lehr , D. P. Schladt , et al., “OPTN/SRTR 2021 Annual Data Report: Lung,” American Journal of Transplantation 23, no. 2 Suppl 1 (2023): S379–S442. 37132345 10.1016/j.ajt.2023.02.009 PMC9970343

[4] C. D. Sanchez‐Hernandez , L. A. Torres‐Alarcon , A. Gonzalez‐Cortes , and A. N. Peon , “Ischemia/Reperfusion Injury: Pathophysiology, Current Clinical Management, and Potential Preventive Approaches,” Mediators of Inflammation 2020 (2020): 8405370. 32410868 10.1155/2020/8405370 PMC7204323

[5] Y. Hu , H. Zhou , H. Zhang , et al., “The Neuroprotective Effect of Dexmedetomidine and Its Mechanism,” Frontiers in Pharmacology 13 (2022): 965661. 36204225 10.3389/fphar.2022.965661 PMC9531148

[6] J. Li , Q. Chen , X. He , et al., “Dexmedetomidine Attenuates Lung Apoptosis Induced by Renal Ischemia‐Reperfusion Injury Through Alpha(2)AR/PI3K/Akt Pathway,” Journal of Translational Medicine 16, no. 1 (2018): 78. 29566706 10.1186/s12967-018-1455-1 PMC5865375

[7] S. Zhang , J. Tang , C. Sun , et al., “Dexmedetomidine Attenuates Hepatic Ischemia‐Reperfusion Injury‐Induced Apoptosis via Reducing Oxidative Stress and Endoplasmic Reticulum Stress,” International Immunopharmacology 117 (2023): 109959. 36881980 10.1016/j.intimp.2023.109959

[8] C. Y. Chiu , S. A. G. Willis‐Owen , K. C. C. Wong , et al., “MAP3K8 Is a Potential Therapeutic Target in Airway Epithelial Inflammation,” Journal of Inflammation (London) 21, no. 1 (2024): 27. 10.1186/s12950-024-00400-2 PMC11264520 39030600

[9] Y. W. Yu , S. Liu , Y. Y. Zhou , et al., “Shexiang Baoxin Pill Attenuates Myocardial Ischemia/Reperfusion Injury by Activating Autophagy via Modulating the ceRNA‐Map3k8 Pathway,” Phytomedicine 104 (2022): 154336. 35849969 10.1016/j.phymed.2022.154336

[10] J. Yue and J. M. Lopez , “Understanding MAPK Signaling Pathways in Apoptosis,” International Journal of Molecular Sciences 21, no. 7 (2020): 2346. 32231094 10.3390/ijms21072346 PMC7177758

[11] A. Awasthi , M. B. Raju , and M. A. Rahman , “Current Insights of Inhibitors of p38 Mitogen‐Activated Protein Kinase in Inflammation,” Medicinal Chemistry 17, no. 6 (2021): 555–575. 32106802 10.2174/1573406416666200227122849

[12] K. Ferenczyova , L. Kindernay , J. Vlkovicova , B. Kalocayova , T. Rajtik , and M. Bartekova , “Pharmacology of Catechins in Ischemia‐Reperfusion Injury of the Heart,” Antioxidants (Basel) 10, no. 9 (2021): 1390. 34573022 10.3390/antiox10091390 PMC8465198

[13] B. Yu , Y. Zhang , T. Wang , et al., “MAPK Signaling Pathways in Hepatic Ischemia/Reperfusion Injury,” Journal of Inflammation Research 16 (2023): 1405–1418. 37012971 10.2147/JIR.S396604 PMC10065871

[14] T. Wang , C. Liu , L. H. Pan , et al., “Inhibition of p38 MAPK Mitigates Lung Ischemia Reperfusion Injury by Reducing Blood‐Air Barrier Hyperpermeability,” Frontiers in Pharmacology 11 (2020): 569251. 33362540 10.3389/fphar.2020.569251 PMC7759682

[15] T. Kong , M. Liu , B. Ji , B. Bai , B. Cheng , and C. Wang , “Role of the Extracellular Signal‐Regulated Kinase 1/2 Signaling Pathway in Ischemia‐Reperfusion Injury,” Frontiers in Physiology 10 (2019): 1038. 31474876 10.3389/fphys.2019.01038 PMC6702336

[16] J. Zhao , G. Wang , K. Han , et al., “Mitochondrial PKM2 Deacetylation by Procyanidin B2‐Induced SIRT3 Upregulation Alleviates Lung Ischemia/Reperfusion Injury,” Cell Death & Disease 13, no. 7 (2022): 594. 35821123 10.1038/s41419-022-05051-w PMC9276754

[17] Y. Si , H. Bao , L. Han , et al., “Dexmedetomidine Attenuation of Renal Ischaemia‐Reperfusion Injury Requires Sirtuin 3 Activation,” British Journal of Anaesthesia 121, no. 6 (2018): 1260–1271. 30442253 10.1016/j.bja.2018.07.007

[18] Z. Liu , J. M. Chen , H. Huang , et al., “The Protective Effect of Trimetazidine on Myocardial Ischemia/Reperfusion Injury Through Activating AMPK and ERK Signaling Pathway,” Metabolism 65, no. 3 (2016): 122–130. 26892523 10.1016/j.metabol.2015.10.022 PMC4967934

[19] Z. Y. Wang , Y. Liu , S. P. Li , et al., “Hypoxia Inducible Factor 1alpha Promotes Interleukin‐1 Receptor Antagonist Expression During Hepatic Ischemia‐Reperfusion Injury,” World Journal of Gastroenterology 28, no. 38 (2022): 5573–5588. 36304082 10.3748/wjg.v28.i38.5573 PMC9594012

[20] N. Haywood , H. Q. Ta , A. Zhang , et al., “Endothelial Transient Receptor Potential Vanilloid 4 Channels Mediate Lung Ischemia‐Reperfusion Injury,” Annals of Thoracic Surgery 113, no. 4 (2022): 1256–1264. 33961815 10.1016/j.athoracsur.2021.04.052 PMC8566328

[21] Y. Mo , R. Wan , L. Feng , S. Chien , D. J. Tollerud , and Q. Zhang , “Combination Effects of Cigarette Smoke Extract and Ambient Ultrafine Particles on Endothelial Cells,” Toxicology In Vitro 26, no. 2 (2012): 295–303. 22178768 10.1016/j.tiv.2011.12.001 PMC3273600

[22] R. Huang , Q. Shi , S. Zhang , et al., “Inhibition of the cGAS‐STING Pathway Attenuates Lung Ischemia/Reperfusion Injury via Regulating Endoplasmic Reticulum Stress in Alveolar Epithelial Type II Cells of Rats,” Journal of Inflammation Research 15 (2022): 5103–5119. 36091334 10.2147/JIR.S365970 PMC9462969

[23] H. Q. Ta , M. Kuppusamy , S. K. Sonkusare , M. E. Roeser , and V. E. Laubach , “The Endothelium: Gatekeeper to Lung Ischemia‐Reperfusion Injury,” Respiratory Research 25, no. 1 (2024): 172. 38637760 10.1186/s12931-024-02776-4 PMC11027545

[24] M. Hou , F. Chen , Y. He , et al., “Dexmedetomidine Against Intestinal Ischemia/Reperfusion Injury: A Systematic Review and Meta‐Analysis of Preclinical Studies,” European Journal of Pharmacology 959 (2023): 176090. 37778612 10.1016/j.ejphar.2023.176090

[25] M. E. Huerter , A. K. Sharma , Y. Zhao , E. J. Charles , I. L. Kron , and V. E. Laubach , “Attenuation of Pulmonary Ischemia‐Reperfusion Injury by Adenosine A2B Receptor Antagonism,” Annals of Thoracic Surgery 102, no. 2 (2016): 385–393. 27109193 10.1016/j.athoracsur.2016.02.060 PMC4958568

[26] D. Lapenna , “Glutathione and Glutathione‐Dependent Enzymes: From Biochemistry to Gerontology and Successful Aging,” Ageing Research Reviews 92 (2023): 102066. 37683986 10.1016/j.arr.2023.102066

[27] L. de Bari , A. Scire , C. Minnelli , L. Cianfruglia , M. P. Kalapos , and T. Armeni , “Interplay Among Oxidative Stress, Methylglyoxal Pathway and S‐Glutathionylation,” Antioxidants (Basel) 10, no. 1 (2020): 19. 33379155 10.3390/antiox10010019 PMC7824032

[28] X. Shi , K. A. Seidle , K. J. Simms , F. Dong , W. M. Chilian , and P. Zhang , “Endothelial Progenitor Cells in the Host Defense Response,” Pharmacology & Therapeutics 241 (2023): 108315. 36436689 10.1016/j.pharmthera.2022.108315 PMC9944665

[29] C. N. Duong and D. Vestweber , “Mechanisms Ensuring Endothelial Junction Integrity Beyond VE‐Cadherin,” Frontiers in Physiology 11 (2020): 519. 32670077 10.3389/fphys.2020.00519 PMC7326147

[30] Q. Guo , M. Ma , H. Yu , Y. Han , and D. Zhang , “Dexmedetomidine Enables Copper Homeostasis in Cerebral Ischemia/Reperfusion via Ferredoxin 1,” Annals of Medicine 55, no. 1 (2023): 2209735. 37162502 10.1080/07853890.2023.2209735 PMC10173798

[31] B. Y. Zhou , J. Yang , R. R. Luo , et al., “Dexmedetomidine Alleviates Ischemia/Reperfusion‐Associated Acute Kidney Injury by Enhancing Autophagic Activity via the alpha2‐AR/AMPK/mTOR Pathway,” Frontiers in Bioscience (Landmark Edition) 28, no. 12 (2023): 323. 38179733 10.31083/j.fbl2812323

[32] D. Wang , L. Wang , J. Han , Z. Zhang , B. Fang , and F. Chen , “Bioinformatics‐Based Analysis of the lncRNA‐miRNA‐mRNA Network and TF Regulatory Network to Explore the Regulation Mechanism in Spinal Cord Ischemia/Reperfusion Injury,” Frontiers in Genetics 12 (2021): 650180. 33986769 10.3389/fgene.2021.650180 PMC8110913

[33] D. Xu , M. L. Matsumoto , B. S. McKenzie , and A. A. Zarrin , “TPL2 Kinase Action and Control of Inflammation,” Pharmacological Research 129 (2018): 188–193. 29183769 10.1016/j.phrs.2017.11.031

[34] J. Tan , D. Liu , X. Lv , et al., “MAPK Mediates Inflammatory Response and Cell Death in Rat Pulmonary Microvascular Endothelial Cells in an Ischemia‐Reperfusion Model of Lung Transplantation,” Journal of Heart and Lung Transplantation 32, no. 8 (2013): 823–831. 10.1016/j.healun.2013.05.005 23747218

[35] Y. Xu , R. Zhang , C. Li , et al., “Dexmedetomidine Attenuates Acute Lung Injury Induced by Lipopolysaccharide in Mouse Through Inhibition of MAPK Pathway,” Fundamental & Clinical Pharmacology 29, no. 5 (2015): 462–471. 26211495 10.1111/fcp.12138

[36] C. C. Lan , C. K. Peng , S. E. Tang , S. Y. Wu , K. L. Huang , and C. P. Wu , “Anti‐Vascular Endothelial Growth Factor Antibody Suppresses ERK and NF‐kappaB Activation in Ischemia‐Reperfusion Lung Injury,” PLoS One 11, no. 8 (2016): e0159922. 27513332 10.1371/journal.pone.0159922 PMC4981443

[37] C. Shi , Z. Li , Y. Wu , et al., “Euscaphic Acid and Tormentic Acid Protect Vascular Endothelial Cells Against Hypoxia‐Induced Apoptosis via PI3K/AKT or ERK 1/2 Signaling Pathway,” Life Sciences 252 (2020): 117666. 32298737 10.1016/j.lfs.2020.117666

[38] S. Xu , Y. Yan , Z. Yan , et al., “Septic Serum Mediates Inflammatory Injury in Human Umbilical Vein Endothelial Cells via Reactive Oxygen Species, Mitogen Activated Protein Kinases and Nuclear Factor‐kappaB,” International Journal of Molecular Medicine 47, no. 1 (2021): 267–275. 33236149 10.3892/ijmm.2020.4785 PMC7723504

[39] B. B. Xue , B. H. Chen , Y. N. Tang , C. W. Weng , and L. N. Lin , “Dexmedetomidine Protects Against Lung Injury Induced by Limb Ischemia‐Reperfusion via the TLR4/MyD88/NF‐kappaB Pathway,” Kaohsiung Journal of Medical Sciences 35, no. 11 (2019): 672–678. 31373750 10.1002/kjm2.12115 PMC11900785

[40] P. B. Dodhiawala , N. Khurana , D. Zhang , et al., “TPL2 Enforces RAS‐Induced Inflammatory Signaling and Is Activated by Point Mutations,” Journal of Clinical Investigation 130, no. 9 (2020): 4771–4790. 32573499 10.1172/JCI137660 PMC7456254

